# Effectiveness of E-Beam Radiation against *Saccharomyces cerevisiae*, *Brettanomyces bruxellensis*, and Wild Yeast and Their Influence on Wine Quality

**DOI:** 10.3390/molecules28124867

**Published:** 2023-06-20

**Authors:** Magdalena Błaszak, Barbara Jakubowska, Sabina Lachowicz-Wiśniewska, Wojciech Migdał, Urszula Gryczka, Ireneusz Ochmian

**Affiliations:** 1 Department of Chemistry, Microbiology and Environmental Biotechnology, West Pomeranian University of Technology in Szczecin, Słowackiego 17 Street, 71-434 Szczecin, Poland; blaszak.magdalena@zut.edu.pl (M.B.); bjakubowska@zut.edu.pl (B.J.); 2 Department of Health Sciences, Calisia University, 4 Nowy Świat Street, 62-800 Kalisz, Poland; 3 Institute of Nuclear Chemistry and Technology, 16 Dorodna Street, 03-195 Warsaw, Poland; w.migdal@ichtj.waw.pl (W.M.); u.gryczka@ichtj.waw.pl (U.G.); 4 Department of Horticulture, West Pomeranian University of Technology Szczecin, Słowackiego 17 Street, 71-434 Szczecin, Poland

**Keywords:** polyphenols, color, yeast, quality of wine, wine preservation, environmental protection

## Abstract

The simplest way to eliminate microorganisms in the must/wine is through sulfuration, as it allows the introduction of pure yeast varieties into the must, which guarantees a high-quality wine. However, sulfur is an allergen, and an increasing number of people are developing allergies to it. Therefore, alternative methods for microbiological stabilization of must and wine are being sought. Consequently, the aim of the experiment was to evaluate the effectiveness of ionizing radiation in eliminating microorganisms in must. The sensitivity of wine yeasts, *Saccharomyces cerevisiae*, *S. cerevisiae* var. *bayanus*, *Brettanomyces bruxellensis*, and wild yeasts to ionizing radiation was com-pared. The effects of these yeasts on wine chemistry and quality were also determined. Ionizing radiation eliminates yeast in wine. A dose of 2.5 kGy reduced the amount of yeast by more than 90% without reducing the quality of the wine. However, higher doses of radiation worsened the organoleptic properties of the wine. The breed of yeast used has a very strong influence on the quality of the wine. It is justifiable to use commercial yeast breeds to obtain standard-quality wine. The use of special strains, e.g., *B. bruxellensis*, is also justified when aiming to obtain a unique product during vinification. This wine was reminiscent of wine produced with wild yeast.. The wine fermented with wild yeast had a very poor chemical composition, which negatively affected its taste and aroma. The high content of 2-methylbutanol and 3-methylbutanol caused the wine to have a nail polish remover smell.

## 1. Introduction

Sulfurization of must eliminates the population of microorganisms during the early stages of vinification. The introduction of pure yeast varieties into the musts assures product quality, due to the well-known metabolic profile of these varieties. The most commonly used wine yeast strain is *Saccharomyces cerevisiae* [1] which ensures a cultivar-specific product due to the repeatability of the chemical composition and the bouquet of the wine [2].

The unique properties of natural and regional wines can be attributed to the compounds produced by unusual microorganisms or hybrids of various wine and beer yeasts. A notable example is Château Musar, which owes its uniqueness to the presence of *Brettanomyces bruxellensis yeast*, contributing to its aromatic profile primarily through 4-ethylphenol and 4-ethylguaiacol. These wines are appreciated by a small group of connoisseurs due to their characteristics of table aroma [3,4].

Natural wines are the result of spontaneous fermentation of grape must, facilitated by a diverse consortium of microorganisms naturally present on the grapes. The predominant wine yeast, *S. cerevisiae*, is involved in the formation of natural wines [5]. The wine yeast *S. cerevisiae* is usually very little in natural musts, just less than 1% of the total population of active microorganisms. Grape must contain a huge biodiversity of microorganisms, including wild yeasts of the genus *Hanseniaspora*, *Candida*, *Metschnikowia*, *Pichia*, *Rhodotorula*, and *Torulaspora*, as well as various bacteria and molds [6]. In recent years, natural wines have become an increasingly important range of wines on the market. They hold great appeal to both connoisseurs and ordinary consumers due to their unique and distinctive qualities [7].

However, spontaneous fermentation does not always produce the expected results. The predominance of wild yeasts that intensely produce higher alcohols can lead to wine spoilage, intense chemical aromas, and vinegary taste sensations. Yeast is responsible for the production of several hundred chemical compounds, and any imbalances can pose risks of unfavorable flavors and aromas [3,5,8].

The addition of sulfur compounds in food products exposes allergies in sensitive consumers [9]. Therefore, alternative methods that do not leave behind preservatives in the final product are being actively pursued. While food irradiation has been commercially employed since the 1950s, the technology is still under development. This applies to the irradiation of new product categories such as wine [9], raw milk [10], as well as lyophilized fruit, vegetables, meat [11] and honey [12].

Furthermore, new areas of application such as irradiation to prevent the spread of pests, especially in the case of tropical fruits and vegetables [13], extending the shelf life of fresh products packed in modified atmospheres [14], and preparing sterilized, shelf-stable food for patients with compromised immune systems or NASA astronauts [15]. One of the significant advantages of food irradiation technology is that it is a nonthermal process, capable of replacing chemical methods or steaming, as demonstrated in the case of dried herbs [16]. Food irradiation is a process of exposing food to ionizing energy to eliminate insects, fungi, or bacteria that can cause human diseases or spoilage, as well as delay the ripening of fresh products. The approved sources of ionizing radiation for food irradiation, as listed in General Standard for Irradiated Foods [17], include gamma rays from radionuclides such as Co-60, X-rays generated from machine sources operating at or below 5 MeV energy level, and electrons generated from machine sources operating at or below 10 MeV energy level.

During the radiation process, microorganisms are effectively killed, and the rip-ening, germination, and spoilage of vegetables are inhibited [18,19]. The effectiveness of ionizing radiation depends on the species of microorganisms, the dose, and the intensity of the radiation [20]. Mold fungi such as *Fusarium oxysporum*, *Phytophthoracitricola*, *Pythium ultimum*, and *Botrytis cinerea* have shown sensitivity to irradiation within the range of 1.5–6 kGy [21]. Three species of *Escherichia coli* O157:H7 suspended in apple juice were sensitive even to a dose of 1 kGy, while complete elimination was achieved at a dose of 2 kGy [20]. Spore-forming bacteria from the genus *Clostridium* and *Enterobacteriaceae* proved resistant to irradiation, with a dose of 4–5 kGy reducing their populations by 90%. Complete elimination of these microorganisms required a minimum dose of 10 kGy [22]. The worldwide development of food irradiation can be attributed to the growing utilization of machine sources of ionizing radiation instead of radioisotopes. Recently, the use of low-energy electrons has gained significant attention. This technology, due to the limited penetration of electrons with energy below 300 keV, is used for surface microbial decontamination [23].

The aim of the experiment was to determine the optimal irradiation dose for the elimination of *S. cerevisiae*, *S. cerevisiae* var. *bayanus*, *B. bruxellensis*, and wild yeasts while ensuring the preservation of wine quality. The analysis encompassed an evaluation of the wine’s chemical composition, color, and organoleptic characteristics.

## 2. Results

**Figure 1:**
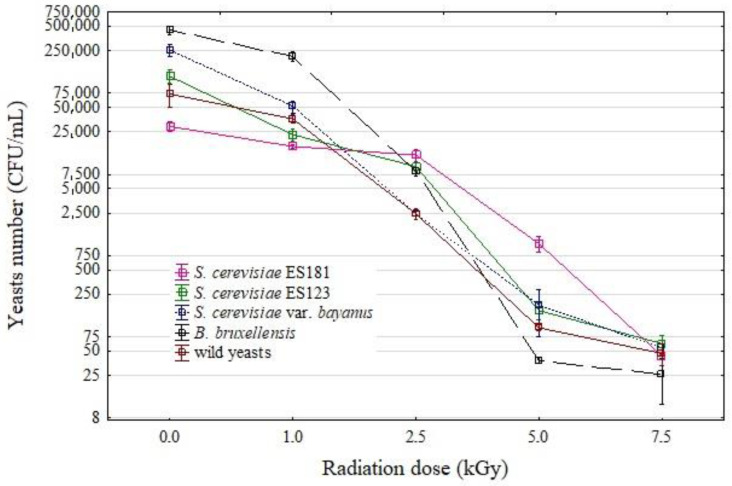
Effect of radiation on the yeast content of wine. The mean and standard deviation are marked.

Four doses of ionizing radiation (1, 2.5, 5.0, and 7.5 kGy) were used to reduce the yeast content in the wine. Upon applying the lowest dose of 1 kGy, a decrease in yeast content was observed (Figure 1). The level of reduction varied among the strains, with *S. cerevisiae* ES181 and *B. bruxellensis* exhibiting greater resistance to the first dose, as their numbers decreased by 40 and 50%, respectively.

In contrast, the strains *S. cerevisiae* ES123 and *S. cerevisiae* var. *bayanus* proved higher sensitivity to irradiation. In these cases, the yeast abundance was nearly 16 and 20%, respectively, relative to the initial value before the application of the physical factor. Notably, a dose of 2.5 kGy significantly reduced the yeast abundance, with only the *S. cerevisiae* ES181 strain remaining at a similar level, representing approximately 60% of the initial value.

The other strains were significantly reduced to levels of a few percent compared to the initial value. Once again, *S. cerevisiae* var. *bayanus* proved the highest sensitivity. A subsequent dose of 5 kGy resulted in nearly complete yeast mortality, with only a 3% reduction observed in the case of *S. cerevisiae* ES181. Finally, a dose of 7.5 kGy left minimal traces of alive yeast cells in each wine sample.

### 2.1. Quality of Irradiated Wine—Selected Parameters

**Figure 2:**
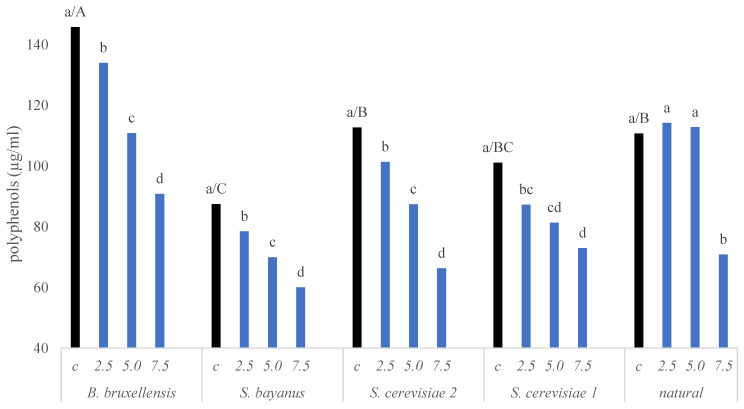
Changes in the content of polyphenolic compounds, depending on the yeast used and the dose of ionizing radiation given. Lowercase letters indicate homogeneous groups within the yeasts and uppercase letters indicate groups between the yeasts.

**Figure 3:**
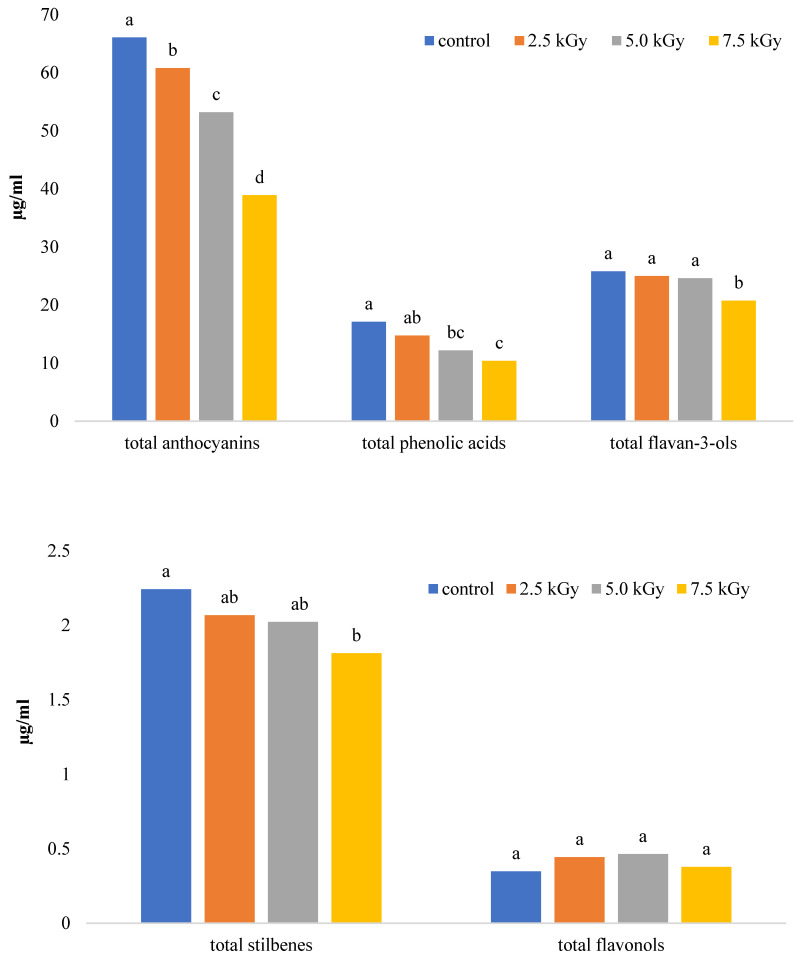
Content of polyphenolic compounds in relation to the ionizing radiation dose. Lowercase letters indicate homogeneous groups within a group of polyphenolic compounds.

**Figure 4:**
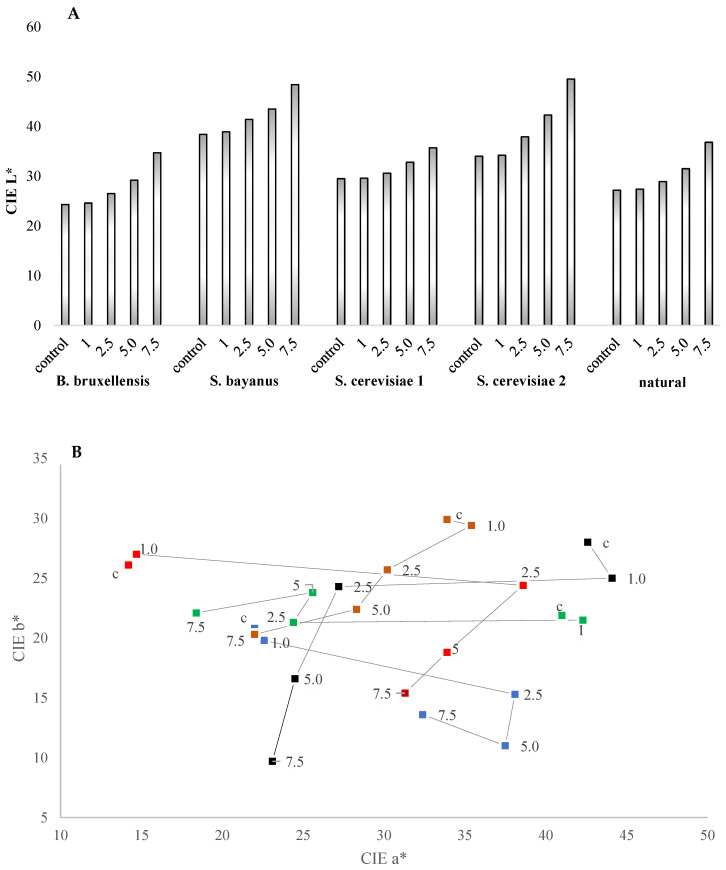
Changes in wine color parameters after the application of ionizing radiation: a monochromatic parameter CIE L* (**A**), chromatic parameter CIE a* and b* (**B**). Types of yeast: *B. bruxellensis*; *S. bayanus*; *S. cerevisiae* ES181; *S. cerevisiae* ES123; wild yeast.

**Figure 5:**
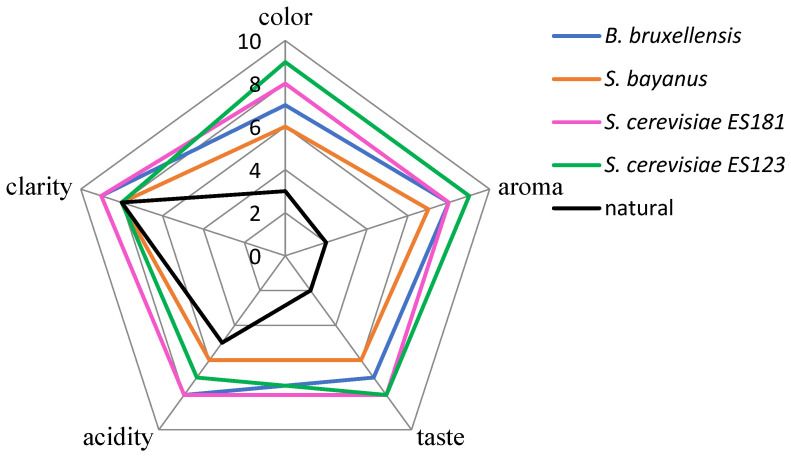
The organoleptic test results.

Regardless of the yeast used, ionizing radiation from a dose of 2.5 kGy already had a negative effect on wine quality. This is indicated by a decreasing polyphenol content and changes in the color of wine (Figure 2, Figure 3 and Figure 4. The most significant changes were observed in anthocyanins and phenolic acids (Figure 3). On the other hand, the lowest dose of 1 kGy had no significant effect on the changes in wine color (Figure 3 and Figure 5), which could be considered advantageous. However, this dose did not achieve a satisfactory reduction in yeast content (Figure 1), rendering its practical use impractical. The higher doses used resulted in a substantial reduction in yeast abundance (Figure 1).

The loss of polyphenols and changes in the color of the wine, characterized by a darkening effect, was observed gradually. With each radiation dose, there was a diminishing presence of polyphenols and red coloring pigments in the wine. A dose of 5 kGy reduced the content of polyphenols by about 20% except for the wine with wild yeast, which exhibited no change in polyphenol content. Anthocyanins and phenolic acids were particularly sensitive to radiation, while other tested polyphenols were moderately sensitive (Figure 3 and Figure 5). Following a dose of 7.5 kGy, the content of polyphenols decreased by approximately 40%. Flavan-3-ols and stilbenes also experienced a reduction of about 20%, while flavonols demonstrated a slight increase, though statistically insignificant (Figure 3).

The changes in polyphenol content and wine color were similar in wines where commercial yeast was used (Figure 2). The polyphenol content experienced a decrease, resulting in lighter and less intensely colored wines. Among the wines subjected to a radiation dose of 7.5 kGy, those inoculated with *S. cerevisiae* ES181 yeast displayed the least reduction in polyphenol content (27.8%), while *S. cerevisiae* 2 wine exhibited the greatest reduction (41.3%). The greatest changes were found in the category of compounds classified as anthocyanins, which are mainly responsible for the color of the wine (Supplementary Table S1).

This observation is further supported by the change in color, as indicated by the L* parameter (Figure 4a). The wines exhibited a lighter shade, and the values of the chromatic color parameters a* and b* decreased (Figure 4b). The smallest changes in the a* and b* color parameter values were observed when the lowest radiation dose of 1 kGy was applied, with values similar to those of nonirradiated wine. In contrast, the most significant changes in color parameters occurred after the subsequent dose of 2.5 kGy.

The greatest color change was observed between these radiation doses, with subsequent doses yielding less pronounced color changes. In the wine produced with natural yeast, even after a radiation dose of 5.0 kGy, the total polyphenol content remained at a similar level to that of untreated wine. However, analysis of individual polyphenolic compounds revealed a decrease in anthocyanin content and an increase in phenolic acids (Supplementary Table S1).

### 2.2. Influence of Yeast Bread on Color, Polyphenol Content, and Selected Chemical Compounds Included in the Wine Aroma

**Figure 6:**
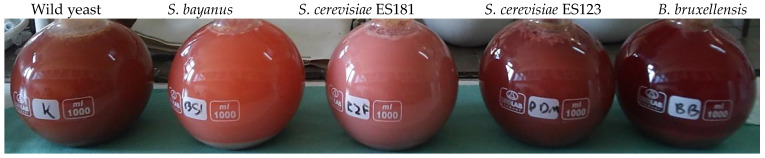
Colors of must/wine after the maceration period.

Four yeast strains, including three strains of *S. cerevisiae* and *B. bruxellensis*, were utilized for fermentation. After fermentation, the wine was analyzed for various parameters, including polyphenols, glycerol, alcohols (isoamyl, iso-butanol, n-propanol, ethyl acetate, isoamyl acetate, isobutyl acetate, acetoin, acetaldehyde, 2 and 3-methylbutanol), and colorrelated factors. The wine treated with *B. bruxellensis* had the highest amount of polyphenols (approximately 140 µg/mL), this wine was also characterized by its dark red color (Figure 2, Figure 5 and Figure 6). The natural wine was also characterized by a high polyphenol content (about 30 µg/mL less than in the wine after using *B. bruxellensis*). In contrast, the wine after using *S. bayanus* had the least polyphenol content and appeared the brightest (Figure 3 and Figure 5a). *S. bayanus* and *S. cerevisiae* ES181 influenced that the wine had the least intense red color.

In the wines examined, a total of 32 polyphenolic compounds were identified and classified into five groups: seven anthocyanins, eight phenolic acids, six flavonols, seven flavan-3-ols, and five stilbenes. Among these compounds, anthocyanins are primarily responsible for the color of pink and red wines. They represented the largest group of polyphenolic compounds in the wines studied (Figure 3). The wines fermented *with B. bruxellensis* exhibited the highest number of anthocyanins (91.78 µg/mL), while the wines with *S. bayanus* had the lowest concentration of anthocyanins (40.31 µg/mL) (Supplementary Table S1).

The contents of the individual compounds in this group varied and depended on the yeast used. Malvidin 3,5-*O*-diglucoside, malvidin 3-*O*-glucoside, and cyanidin 3,5-*O*-diglucoside (Supplementary Table S1) were found to be the highest in all wines. In red grape cultivars, malvidin derivatives can contribute up to 85% of all anthocyanins [24]. The content of flavan-3-ols in the wines was relatively unaffected by the yeast used, ranging from 23 to 29 µg/mL. Despite the high polyphenol content, wines fermented with wild yeast exhibited the lowest levels of phenolic acids (6.33 µg/mL). In the other wines, the content of these compounds ranged from 16.37 to 22.43 µg/mL. Flavonols and stilbenes were the smallest groups of compounds present in the wines.

The highest amount of glycerol was present in natural wine (5.3 g/L) and the wine made after using *B. bruxellensis* and significantly less in wines with *S. cerevisiae* strains (Table 1). The least amount of higher alcohols were found in wines after the use of *S. cerevisiae* yeast (about 130 mg/L on average), while more than twice as much was found in natural wine and the wine after the use of the *B. bruxellensis* strain (Table 1). Isoamyl alcohol was highest in all wines, ranging from 88 to 252 mg/L (*S. cerevisiae* 2 and wild yeast, respectively). Propanol and isobutanol were also present in the highest concentrations in natural wines and wines containing *B. bruxellensis.*

According to Table 1, aryl alcohols (the sum of 2-methylbutanol and 3-methylbutanol) were highest in natural wine, with a concentration of about 100 mg/L, while wines made with classical *S. cerevisiae* strains had lower levels, averaging around 69 mg/L. Acetaldehyde content varied depending on the yeast used, ranging from approximately 29 (*S. cerevisiae* 2) to 63 mg/L (wild yeast), with the highest concentration observed in *B. bruxellensis* wine at 67 mg/L. Ethyl acetate was most abundant in wine produced with wild yeast (53.2 mg/L), but high levels were also found in wine fermented with *B. bruxellensis* (47.1 mg/L). *S. cerevisiae* wines also contained ethyl acetate and acetaldehyde, but at lower levels compared to the other yeast strains.

The blind organoleptic evaluation of the wines showed that all wines received high scores in terms of clarity (Figure 5). However, the other parameters, such as taste and aroma, were strongly influenced by the breed of yeast used. The wine made with wild yeast received the lowest rating, particularly in terms of taste and aroma. This is consistent with its chemical composition and high content of undesirable substances, as shown in Table 1. The wines fermented with *S. cerevisiae* yeast were rated the highest in overall quality.

**Table 1:** Fermentation byproducts.

Fermentation Byproducts	*B. bruxellensis*	*S. bayanus*	*S. cerevisiae* 1 (ES 181)	*S. cerevisiae* 2 (ES 123)	Wild Yeasts
Isoamyl (mg/L)	177 d	126 c	88 a	97 b	252 e
Iso-butanol (mg/L)	40.1 e	26.3 c	23.0 b	19.6 a	35.5 d
N-propanol (mg/L)	52.3 c	28.9 b	18.7 a	16.2 a	78.6 d
Total	269.4 C	181.2 B	129.7 A	132.8 A	366.1 D
Glycerol (g/L)	4.53 d	4.28 c	3.90 b	2.81 a	5.34 e
Ethyl acetate (mg/L)	47.1 c	37.8 b	26.5 a	22.9 a	53.2 d
Isoamyl acetate (mg/L)	0.18 c	0.14 b	0.13 ab	0.11 a	0.22 d
Isobutyl acetate (mg/L)	0.55 cd	0.49 bc	0.68 de	0.72 e	0.24 a
Acetoine (mg/L)	0.31 cd	0.35 d	0.12 a	0.17 b	0.27 c
Acetaldehyde (mg/L)	67.4 d	49.7 c	33.5 b	29.6 a	63.0 d
2 and 3-Methyl-butanol (mg/L)	98.4 c	78.4 b	66.8 a	72.5 ab	112.2 d

Explanation: Mean values denoted by the same letter do not differ statistically significantly at 0.05, according to the t-Tukey test.

## 3. Discussion

### 3.1. Effects of Ionizing Radiation on Yeast and Polyphenols

In a study by Błaszak et al. [9], ionizing radiation at a dose of 1 kGy reduced the number of yeast by approximately 95%, and complete elimination of yeast was observed after the application of 2.5 kGy. Similarly, in the experiment presented here, 1 kGy significantly reduced yeast viability, with between 40 and 85% remaining (depending on the yeast strains used). The radiation dose of 2.5 kGy had a significant effect on reducing yeast abundance, with only a few percent of yeast remaining compared to the control wine. The exception was with the strain *S. cerevisiae* ES181, as radiation at a dose of 2.5 kGy only reduced the number of these yeasts by 40%, with minimal impact on the degradation of polyphenols. Therefore, based on the results of Błaszak et al. [9] and those presented in this work, a radiation dose of 2.5 kGy is the best option for wine preservation. This dose effectively kills yeast while preserving phenols.

Electron beam irradiation affected the degradation of tannins in the species, simultaneously increasing the content of phenolic acids. Additionally, the changes in anthocyanin content within the wine corresponded to a change in its color. Morata et al. [25] also observed a decrease in polyphenol content and color changes in wine exposed to a higher dose of irradiation.

The electron beam irradiation of spices affected the degradation of tannins while increasing the content of phenolic acids. Phenolic acids underwent degradation only after the application of a high dose of radiation—10 kGy [26]. The change in anthocyanin content in this wine also manifested as a change in wine color. The wine became brighter, which may indicate its oxidation. The degradation products of these compounds caused by radiation or the formation of free radicals may be formed [27]. Morata et al. [25] also observed a decrease in polyphenol content and changes in wine color after subjecting it to higher radiation doses.

### 3.2. Influence of Yeast on the Bouquet of Wine

In line with the Georgian/natural/homegrown wine tradition, some winemakers have started to explore alternative yeast strains beyond the standard *Saccharomyces* for wine production. These wild yeasts contribute to spontaneous fermentation, imparting unique flavors and aromas to the wines [28]. However, non *Saccharomyces* yeasts produce various metabolites that can worsen the quality of the wine, including undesirable flavors such as acetic acid, acetoin, ethyl acetate, and acetaldehyde, as well as off-putting aromas associated with vinyl phenols and ethyl phenols, which are linked to the development of *Brettanomyces/Dekkera* yeast strains. The use of such yeast raises concerns regarding both the safety and validity of their application [29,30].

During the process of alcoholic fermentation, glycerol emerges as the third most quantitatively produced compound, following ethanol and carbon dioxide. Its content ranges from 1 to 15 g/dm^3^ [31]. Glycerol is odorless and therefore has no effect on the aroma of the wine. However, owing to its thick and oily texture, it influences the flavor of the wine, imparting a smooth, velvety aftertaste, and enhancing the overall richness and fullness of flavor. Glycerol concentrations tend to be considerably higher in red wines compared to white wines. The sensory threshold for glycerolize, the perception of glycerol, has been established at 5.2 g/dm^3^ [32,33].

The level of glycerol in wine products depends on many factors, including the type of yeast strain employed, sugar levels, must pH, SO_2_ content, and fermentation temperature [34]. In our study, all wines were produced from the same must under identical fermentation conditions, maintaining a temperature of 12 ± 0.5 °C. The only variable was the breed of yeast strain. Remarkably, the wine fermented with wild yeast exhibited the highest glycerol content, as indicated in Table 1). The high glycerol content in the wines gave pronounced teardrops, which are characteristic forms formed on the walls of the glass when droplets flow back into the glass. Wines produced with *S. cerevisiae* had the least amount of glycerol.

One of the main groups of compounds synthesized by yeast is higher alcohols, also known as fusel alcohols. These alcohols exhibit an intense aroma that plays an important role in shaping the bouquet of wine. At low concentrations (below 300 mg/dm^3^), they have a positive effect on the aroma, while higher concentrations can mask the inherent aroma of the beverage [29].

Only the wine produced with wild yeast exceeded levels of 300 mg/L for these alcohols. This confirms the opinion that high concentrations can be unfavorable, as this wine received the worst judgment from testers in terms of both aroma and taste (Figure 1 and Figure 2). Once again, wines made with *S. cerevisiae* yeast had the least amount of these alcohols (Table 1). Among these alcohols, isoamyl alcohol was highest in all wines, ranging from 88 to 252 mg/L (*S. cerevisiae* 2 and wild yeast, respectively).

Only the wine produced with wild yeast exceeded 300 mg/L of these alcohols. This confirms the opinion that high concentrations can be unfavorable, as this wine received the worst judgment from testers in terms of both aroma and taste (Figure 1 and Figure 2). Once again, wines made with *S. cerevisiae* yeast had the least amount of these alcohols (Table 1). Among these alcohols, isoamyl alcohol was highest in all wines, ranging from 88 to 252 mg/L (*S. cerevisiae* 2 and wild yeast, respectively).

According to other authors [35], isoamyl alcohol usually is above 70% of the total of these compounds, with a content range of 12–310 mg/L. In the wines studied, it ranged from 67 to 73%. The presence of propanol in wines is typically between 10 and 125 mg/dm^3^, while isobutanol ranges from 15 to 175 mg/L [1,35].

In the wines studied, these alcohols were found at the lower end of these ranges. The amount of fusel alcohols produced during fermentation is significantly influenced by the breed of yeast while reducing their concentration can be achieved by using nitrogenous fermentation media [36].

Apart from isobutanol, another compound that has a particularly negative effect on the sensory properties of fermented beverages is aryl alcohols (the sum of 2 and 3-methylbutanol). They contribute to an unpleasant solvent-like aroma and taste [37]. Once again, wines fermented with wild yeast were characterized by the highest content of these compounds.

Aldehydes present in grapes play a crucial role in the formation of cultivar aromas, and their levels in young wines typically do not exceed 75 mg/L. Among this group of compounds, acetaldehyde stands out as the most significant. In our study, its concentration ranged from 29.6 (*S. cerevisiae* 2) to 63 mg/L (wild yeast) and 67.4 mg/L (*B. bruxellensis*). Acetaldehyde plays a vital role in the stabilization of wines, especially red wines, during aging. It accelerates the polymerization reaction of anthocyanins and phenols, contributing to the overall quality and structure of the wine [38].

Ethyl acetate, when present in low concentrations, enhances the fruity aromas in beverages, such as pear, peach, pineapple, and raspberry. However, at higher concentrations, it can develop an odor reminiscent of varnish or nail polish remover. The acetic acid esters that contribute to aroma are isoamyl acetate—banana and isobutyl acetate—fruit [39]. Once again, the wine fermented with wild yeast displayed notable distinctions in terms of these compounds. Isoamyl acetate exhibited the highest concentration in this wine, while isobutyl acetate demonstrated the lowest concentration.

Acetoin affects the buttery aroma of wine and is produced by lactic acid bacteria (*Oenococcus oeni*) from citric acid, thereby enhancing the buttery aroma of the wine [40]. The presence of yeast cultures other than *Saccharomyces* can influence the production of various metabolites that affect wine quality, including acetoin, ethyl acetate, and acetaldehyde, as well as compounds associated with unpleasant odors such as ethyl phenols, which are associated with the development of Brettanomyces/Dekkera [41,42]. Our study aligns with these findings, as wines fermented with *S. cerevisiae* exhibited lower levels of acetoin, as well as ethyl acetate and acetaldehyde (Table 1).

The fermentation process with *B. bruxellensis* yeast was observed to be the fastest and most rapid compared to other inoculated yeasts. The rapid fermentation may have increased the ethanol content, facilitating compounds’ extraction from the fruit. The fastest change in extract content was observed. Notably, this particular wine displayed the highest polyphenol content (Figure 3) and had the darkest color (Figure 4). In contrast, wines fermented with *S. bayanus* yeast had the least polyphenols and appeared brightest. These wines also characterized the lowest values for the a* and b* color parameters (Figure 4). In some fermentation processes, such as beer production, the presence of *B. bruxellensis* can be considered beneficial, as this strain contributes to the specific characteristics and aromas of these specialty beverages. Likewise, unique wines that owe their specific aroma to compounds like 4-ethylphenol, 4-ethylguaiacol, and tetrahydropyridine produced by *B. bruxellensis* also find connoisseurs [43,44].

The fermentation process of wine with wild yeast can also be influenced by the cultivation method. In our experiment, the fruit was harvested from an organic plantation, where there was/may have been greater microbial biodiversity. The must on wild yeast fermented rapidly and turbulently. The polyphenol content is comparable to wines made by *S. cerevisiae* and higher than in wines made by *S. bayanus*.

An organoleptic evaluation of the five parameters performed blind showed that all wines scored highly only in terms of clarity (Figure 5). However, the other parameters were strongly influenced by the breed of yeast used. By far the lowest rating was given to the wine made with wild yeast. In particular, taste and aroma were rated very low. This was confirmed by its chemical composition and high content of undesirable substances (Table 1). The wines in which *S. cerevisiae* yeast was used were rated best. Spontaneous fermentation has a beneficial effect when carried out in vineyards that have relied on indigenous wild yeast strains for generations.

Over the years or even decades, a distinctive and unique microbiome is produced in the vineyard, and so fermentation also has a predictable course to a certain extent. Such natural wines are of great interest to connoisseurs and are very expensive [45].

Apart from possessing strong antioxidant properties, polyphenols also influence the sensory characteristics of food products [46]. The polyphenol content and the colour of the wine were decisively influenced by the yeast breeds used (Figure 3 and Figure 4). The yeasts used during alcoholic fermentation have a strong influence on the rate of alcohol production. During fermentation from the skins, extraction of the constituents in the skins also occurs [29]. It was observed that the process of turbulent fermentation was the fastest and most rapid in most inoculating with *B. bruxellensis* yeast. The rapid fermentation may have increased the ethanol content, facilitating compounds’ extraction from the fruit.

The fastest change in extract content was observed in this setting/ must/wine. This wine had the highest polyphenol content (Figure 3) and was the darkest (Figure 4). The wine fermented with *S. bayanus* yeast was the least polyphenols and the lightest. This wine also had the lowest a* and b* color parameters (Figure 4).

In the wines studied, 32 polyphenolic compounds were identified and classified into 5 groups: 7 anthocyanins, 8 phenolic acids, 6 flavonols, 7 flavan-3-ols and 5 stilbenes. In rosé and red wines, anthocyanins are mainly responsible for the colour. They constituted the largest group of polyphenolic compounds in the wines studied (Figure 3). Their content reflects the content of total polyphenols. They were highest in wines fermented with *B. bruxellensis* (91.78 µg/mL) and lowest in wines with *S. bayanus* (40.31 µg/mL) (Supplementary Table S1). The content of the individual compounds in this group varied and depended on the yeast used.

Malvidin 3,5-*O*-diglucoside, malvidin 3-*O*-glucoside and cyanidin 3,5-*O*-diglucoside (Supplementary Table S1) were the most abundant in all wines. In red grape cultivars, malvidin derivatives may account for as much as 85% of all anthocyanins [24]. Flavan-3-ols were another group of compounds, but their content in wine was little affected by yeast (23–29 µg/mL). Despite the high polyphenol content, wines with wild yeast had the least phenolic acids (6.33 µg/mL). In the other wines, the content of these compounds ranged from 16.37 to 22.43 µg/mL. Flavonols and stilbenes were the smallest groups of compounds.

The fermentation process of wine with wild yeast can also be influenced by the cultivation method. In our experiment, the fruit was harvested from an organic plantation, where there was/may have been greater microbial biodiversity. The must on wild yeast fermented rapidly and turbulently. The polyphenol content is comparable to wines made on *S. cerevisiae* and higher than those made on *S. bayanus*. In the experiment of Sterczyńska et al. [47], the fruit was harvested from a conventional plantation. The fermentation process on wild yeast was slower, the wine had less alcohol and polyphenols compared to wine made on *S. cerevisiae*.

## 4. Materials and Methods

### 4.1. Characteristics of the Area of Research and Plant Material

The grape was harvested at the research station West Pomeranian University of Technology in Szczecin located in the north-western part of Poland. The majority of the West Pomeranian Province belongs to the 7A zone on Heinz and Schreiber’s “Map of zones of plant resistance to frost”. However, in the area of Szczecin and in the nearby northern region, minimal temperatures range from −12 °C to −15 °C, which corresponds to values typical of zone 7B. The average temperature during the growing season (April-October) between 1951 and 2012 was 13.7 °C and rainfall was 391 mm [48]. The soil in the vineyard was agricultural soil with a natural profile, developed from silt-loam, pH 6.9 higher water capacity and optimal mineral content [49].

The vines were grafted on SO_4_ rootstock and planted in 2016 with a North-South row orientation at 1.01 m × 2.18 m. The vines were pruned with a Guyot (one arm) training system and vertically positioned with eight shoots, each had two clusters. Standard vineyard management methods for organic plantations were used in both growing seasons.

### 4.2. Description of the Variety and Production of Wine

The study involved the dark-skinned vine cultivar Souvignier Gris, which is a German cultivar with increasing interest in its cultivation in cool climate areas. The vine is valued especially due to its high fungus- and cold-resistant. ‘Souvignier Gris’ is a cross between the grapes Cabernet Sauvignon and Bronner. Berries of ‘Souvignier Gris’ were harvested in October (25.4 Brix) and immediately crushed in order to prepare grape must. Grape must be inoculated with commercial yeast or left to wild yeast. The dry active wine yeast (30 g/hL) was prepared with 150 mL of water at 35 °C and added to the grape must.

The wine was prepared in steel containers with a volume of 50 L. The fruits were separated from the stalks, crushed and then macerated for 3 days at 14 °C. Then they must be pressed on the wine press. The experiment with e-beam irradiation of the wine was performed four weeks after the initiation of alcoholic fermentation.

### 4.3. Yeast: Assessment of Their Numbers in the Wine

The test yeast, *S. cerevisiae* (ES181; ES 123—ES Viniarske Potrebys.r.o.), and *S. cerevisiae* var. *bayanus* (Fermivin LS2—Brovin) are strains that are widely used for the vinification of grape musts on the industrial scale. These yeast strains are characterized by high tolerance of the alcohol content (16.5%) and sugar content (300 g/L) in the culture medium. *B. bruxellensis* (WLP650—White Labs) was also used in the experiments. Inoculation was carried out 12 h after the wine decontamination process was performed. Radiation was applied at doses of 1.0, 2.5, 5.0, and 7.5 kGy. The yeast counts in the wine were recorded in accordance with ISO 21527–1:2008 [50]. A specialized yeast culture medium was used—YPG Agar (Sigma-Aldrich, Melbourne, Australia). Wine samples were taken after serial decimal dilutions and were then added to the microbial medium (deep inoculation) and then incubated for 3 days at 25 °C; the colony-forming units (CFU) were then counted using the eCount Colony Counter (AllChem, Beirut, Lebanon). The experiments were performed in triplicate.

### 4.4. Irradiation

The Institute of Nuclear Chemistry and Technology (Warsaw, Poland) has unique devices and elaborate procedures for the process of irradiation, ensuring high efficiency of sterilization and microbiological decontamination. The Accelerator ELEKTRONIKA 10/10 is a high-power radiation device that allows electron beams with 9 MeV energy and average power of up to 10 kW to be obtained. These parameters allow the irradiation process to be performed at a commercial scale. The main parameters of the ELEKTRONIKA 10/10 accelerator: are pulse electron beam mode; electron energy of 8–10 MeV; average beam power of 10 kW; dose rate of 700 Gy/s.

The linear electron accelerator Elektronika 10-10 was used for wine irradiation with doses in the range of 1.0–7.5 kGy (in triplicate). The defined doses were controlled using graphite calorimeters from RISO High Dose Reference Laboratory, in Denmark. For the determination of electron energy and dose uniformity RISO B3 dosimetric foil was measured with a flatbed scanner and RisoScan software was used. The uncertainty of dose measurements was about 10% due to the dosimetric system and the instability of irradiation conditions. The electron energy used for the irradiation of yeasts in wine was 9 MeV. Wine samples were packed in 5 mL plastic tubes. Each tube was placed horizontally to the beam to ensure as high as possible dose uniformity, determined as dose maximum to dose minimum ratio. In the conditions of the experiments, the dose uniformity was 1.1. The doses presented are the average doses calculated based on the depth dose profile of the beam in water.

### 4.5. Color Measurement

The color parameters were L* (L* = 100 indicates white; L* = 0 indicates black), a* (+a* indicates redness; −a* indicates greenness), b* (+b* indicates yellow; −b* indicates blue). Color coordinates were determined in the CIE L*a*b* space for the 10° standard observer and the D 65 standard illuminant. CIE L*a*b* was measured using a Konica Minolta CM-700d spectrophotometer [49].

### 4.6. Identification of Compounds in Wine

#### 4.6.1. Phenolics

Identification and quantification of the polyphenol values of the extracts were carried out using an ACQUITY Ultra Performance LC system, equipped with a photodiode array detector with a binary solvent manager (Waters Corporation, Milford, MA, USA) and a mass detector equipped with an electrospray ionization (ESI) source operating in negative and positive modes, as described by Oszmiański et al. [51]. Separation of the individual polyphenols was carried out using a UPLC BEH C18 column (1.7 m, 2.1 × 100 mm, Waters Corporation, Milford, MA, USA) at 30 °C. The samples (10 L each) were injected, and then the elution was completed in 15 min with a sequence of linear gradients and isocratic flow rates of 0.45 mL/min The mobile phase consisted of solvent A (2.0% formic acid, *v*/*v*) and solvent B (100% acetonitrile). The program began with isocratic elution using 99% of solvent A (0–1 min), then a linear gradient was used until the 12-min point, lowering solvent A to 0%; from 12.5 to 13.5 min, the gradient returned to the initial composition (99% A), then it was maintained at a constant level to re-equilibrate the column. The analysis was carried out using full-scan, data-dependent MS scanning from *m*/*z* 100 to 1500. Leucine enkephalin was used as the reference compound at a concentration of 500 pg/L and at a flow rate of 2 L/min; the [M−H]^−^ ion was detected at 554.2615 Da. The [M−H]^−^ ion was detected during a 15-min analysis performed within ESI–MS accurate mass experiments, which were permanently introduced via the Lock Spray channel using a Hamilton pump. The lock mass correction was ± 1.00 for the mass window. The mass spectrometer was operated in negative- and positive-ion modes, set to the base peak intensity (BPI) chromatograms, and scaled to 12,400 counts per second (cps) (100%). The optimized MS conditions were as follows: capillary voltage of 2500 V; cone voltage of 30 V, source temperature of 100 °C; desolvation temperature of 300 °C; desolvation gas (nitrogen) flow rate of 300 L/h. Collision-induced fragmentation experiments were performed using argon as the collision gas, with voltage ramping cycles from 0.3 to 2 V. Characterization of the single components was carried out via the retention time and the accurate molecular masses. Each compound was optimized to its estimated molecular mass [M−H]^−^/[M+H]^+^ in the negative and positive modes before and after fragmentation. The data obtained from UPLC–MS were subsequently entered into the MassLynx 4.0ChromaLynx Application Manager software. On the basis of these data, the software is able to scan different samples for the characterized substances. The runs were monitored at the wavelength for flavonol glycosides of 360 nm. The PDA spectra were measured over the wavelength range of 200–800 nm, in steps of 2 nm. The retention times and spectra were compared to those of the pure standard [51].

#### 4.6.2. Glycerol

Analyses were performed using a Shimadzu NEXE-RA XR instrument (Kyoto, Japan) with an RF-20A refractometric detector. Separation was carried out on an Asahipak NH2P-50 250 × 4.6 mm Shodex column (Showa Denko Europe, Munich, Germany). For quantitative measurements, the standard curves prepared for the glycerol standard were used.

#### 4.6.3. Volatile Compound Analysis

Qualitative analysis of the volatile aromas of the two highest-scoring wood-aging wines was carried out using the gas chromatography-mass spectrometer (GC-SPME) technique, using a device equipped with a flame ionization detector (FID) and a DB-WAX capillary column, with helium as a carrier gas. The oven temperature was kept at 40 °C for 7 min, followed by an increase to 230 °C at a rate of 3 °C/min.

### 4.7. Sensory Evaluation

The wines were subjected to sensory evaluation. A group comprising 35 tasters evaluated the quality of the wine. Before starting the sensory evaluation of the wines, the testers were trained and informed about the purpose of the assessment. The people who made the assessment were not professional testers. Wine samples (30 mL) were evaluated in 100 mL wine glasses. Color, aroma, flavor, acidity, and clarity were assessed on a scale of 1 to 10 (with 10 being the best score for a given characteristic). The arithmetic mean for each trait of wine quality was calculated on the basis of individual assessments and a chart was developed.

### 4.8. Statistical Analysis

All statistical analyses were performed using the Statistica 12.5 software (StatSoft Polska, Cracow, Poland). The data were then subjected to one-factor variance analysis (ANOVA). Mean comparisons were performed using Tukey’s least significant difference (LSD) test; significance was set at *p* < 0.05.

## 5. Conclusions

Ionizing radiation effectively eliminated the yeast in the wine. The optimal dose was determined to be 2.5 kGy, resulting in a reduction of yeast population by over 90%, except for one strain. Importantly, the quality of the wine was not significantly reduced. Radiation applied at higher doses worsened the organoleptic and chemical properties of the wine. As the radiation dose increased, the yeast in the wine progressively and rapidly declined. The differences in yeast response are related to the extent of radiation exposure.

Among the yeast breeds studied, only the *S. cerevisiae* ES181 strain exhibited rela-tively higher resistance to radiation doses of 1 and 2.5 kGy. In contrast, the other yeast breeds were subject to reduction, starting from 1 kGy, although to varying degrees. Therefore, it is reasonable to conduct a pretest to determine the sensitivity of the yeasts being used in the different levels of reduction.

In general, it can be said that the breed of yeast used has a very strong influence on the quality of the wine. It is justifiable to use commercial yeast breeds to obtain standard-quality wines. However, there are instances where the use of special yeast strains, such as *B. bruxellensis*, can be justified if the aim of vinification is to deliver or produce. *B. bruxellensis* strains rapidly fermented the sugars in the must, resulting in higher alcohol content. This extraction of alcohol contributes to the intensity of the red color in the wine, as it interacts with the anthocyanins.

## Data Availability

Not applicable.

## Supplementary Materials

The following supporting information can be downloaded at: https://www.mdpi.com/article/10.3390/molecules28124867/s1, Table S1: The content of polyphenols [μg/mL].
