# Supplementary material for: Effectiveness of E-Beam Radiation against Saccharomyces cerevisiae, Brettanomyces bruxellensis, and Wild Yeast and Their Influence on Wine Quality
Source: Molecules. 2023 Jun 20;28(12):4867. doi: 10.3390/molecules28124867 (PMC10301072; doi:10.3390/molecules28124867)
Supplement: Supplementary file 1 [file molecules-28-04867-s001.zip › molecules-2350622-supplementary.pdf]

Tabela 1S. The content of polyphenols [ug/mL]

**Compounds**	***B. bruxellensis***					***S. bayanus***					***S. cerevisiae* 1 (ES 181)**					***S. cerevisiae* 2 (ES 123)**					**Wild Yeasts**				
	**Control**	**2.5**	**5**	**7.5**	**Mean**	**Control**	**2.5**	**5**	**7.5**	**Mean**	**Control**	**2.5**	**5**	**7.5**	**Mean**	**Control**	**2.5**	**5**	**7.5**	**Mean**	**Control**	**2.5**	**5**	**7.5**	**Mean**
Cyanidin 3,5-*O*-diglucoside	17.67	16.88	14.94	11.22	**15.18**	7.6	7.41	6.04	4.98	**6.51**	14.9	13.91	12.72	8.44	**12.49**	12.26	10.5	9.07	8.47	**10.08**	17.21	16.34	12.93	9.45	**13.98**
Delphinidin 3-*O*-glucoside	9.61	9.58	8.38	5.25	**8.21**	5.48	5.52	4.83	2.59	**4.61**	6.66	6.02	4.81	2.56	**5.01**	7.32	7.24	6.02	5.18	**6.44**	11.96	11.83	10.91	7.06	**10.44**
Delphinidin 3,5-diglucoside	7.94	6.65	6.12	3.69	**6.10**	3.73	3.11	2.86	2.20	**2.98**	8.90	8.64	6.53	4.89	**7.24**	5.66	5.05	5.24	3.24	**4.80**	5.00	4.64	4.00	3.77	**4.35**
Malvidin 3,5-*O*-diglucoside	26.47	25.42	19.77	16.24	**21.98**	9.79	8.96	7.86	6.03	**8.16**	8.40	6.89	6.16	4.36	**6.45**	9.49	6.70	6.02	5.52	**6.93**	15.98	15.77	14.07	9.32	**13.79**
Malvidin 3-*O*-glucoside	17.52	17.01	16.21	11.5	**15.56**	8.24	8.02	7.86	4.47	**7.15**	14.29	12.91	11.85	7.94	**11.75**	12.49	11.99	10.88	9.19	**11.14**	16.09	13.58	12.88	6.65	**12.30**
Peonidin 3-*O*-glucoside	3.95	3.17	2.79	1.25	**2.79**	1.85	1.70	1.27	1.39	**1.55**	6.59	4.19	3.56	4.92	**4.82**	2.81	2.73	1.97	1.73	**2.31**	4.91	4.40	2.75	2.95	**3.75**
Petunidin 3-*O*-glucoside	8.62	7.84	7.00	5.02	**7.12**	3.62	2.97	2.35	1.03	**2.49**	4.43	4.16	3.27	2.41	**3.57**	5.94	5.43	4.97	3.32	**4.92**	6.82	6.78	7.02	6.19	**6.70**
**Anthocyanins total**	**91.78**	**86.55**	**75.21**	**54.17**	**76.92**	**40.31**	**37.69**	**33.07**	**22.69**	**33.44**	**64.17**	**56.72**	**48.9**	**35.52**	**51.32**	**55.97**	**49.64**	**44.17**	**36.65**	**46.60**	**77.97**	**73.34**	**64.56**	**45.39**	**65.31**
Gallic acid	0.01	0.12	0.19	0.05	**0.09**	0.05	0.05	0.09	0.06	**0.06**	0.04	0.29	0.54	0.62	**0.37**	0.2	0.44	0.03	0.14	**0.20**	0.05	0.03	0.03	0.02	**0.03**
Protocatechuic acid	1.39	1.29	1.09	0.75	**1.13**	1.51	1.29	1.17	0.67	**1.16**	1.77	1.55	1.41	1.16	**1.47**	1.39	1.20	1.12	1.25	**1.24**	0.99	1.20	1.51	0.75	**1.11**
Caftaric acid	10.69	8.33	3.60	3.13	**6.44**	8.79	6.57	4.43	5.49	**6.32**	9.05	7.80	4.45	4.33	**6.41**	7.34	4.51	4.76	5.38	**5.50**	2.77	5.03	8.93	3.42	**5.04**
Coutaric acid	9.72	7.92	3.90	3.88	**6.36**	8.93	6.50	4.38	6.49	**6.58**	9.49	6.65	4.06	4.11	**6.08**	7.20	4.27	5.06	5.36	**5.47**	2.34	6.98	8.82	3.69	**5.46**
Caffeic acid	0.13	0.15	0.09	0.08	**0.11**	0.15	0.13	0.11	0.09	**0.12**	0.08	0.08	0.08	0.05	**0.07**	0.06	0.05	0.11	0.04	**0.07**	0.10	0.11	0.06	0.05	**0.08**
p-Coumaric acid	0.26	0.24	0.15	0.18	**0.21**	0.16	0.09	0.05	0.07	**0.09**	0.10	0.06	0.04	0.04	**0.06**	0.06	0.03	0.09	0.04	**0.06**	0.03	0.10	0.06	0.04	**0.06**
Coumaric acid	0.22	0.16	0.11	0.13	**0.16**	0.18	0.14	0.10	0.10	**0.13**	0.13	0.09	0.06	0.06	**0.09**	0.11	0.07	0.12	0.03	**0.08**	0.05	0.15	0.14	0.17	**0.13**
Ferulic acid	0.01	0.01	0.01	0.01	**0.01**	0.01	0.00	0.01	0.00	**0.01**	0.00	0.01	0.00	0.00	**0.00**	0.01	0.00	0.00	0.00	**0.00**	0.00	0.00	0.01	0.00	**0.00**
**Phenolic acids total**	**22.43**	**18.22**	**9.14**	**8.21**	**14.5**	**19.78**	**14.77**	**10.34**	**12.97**	**14.465**	**20.66**	**16.53**	**10.64**	**10.37**	**14.55**	**16.37**	**10.57**	**11.29**	**12.24**	**12.61**	**6.33**	**13.6**	**19.56**	**8.14**	**11.90**
Myricetin-3-*O*-glucoside	0.10	0.27	0.10	0.15	**0.16**	0.22	0.13	0.2	0.24	**0.20**	0.12	0.27	0.29	0.19	**0.22**	0.11	0.1	0.19	0.08	**0.12**	0.08	0.1	0.08	0.07	**0.08**
Myricetin-3-*O*-rutinoside	0.01	0.02	0.02	0.03	**0.02**	0.02	0.01	0.02	0.01	**0.02**	0.02	0.02	0.02	0.01	**0.02**	0.01	0.01	0.03	0.01	**0.02**	0.01	0.01	0.01	0.01	**0.01**
Quercetin 3-*O*-rutinoside	0.00	0.00	0.00	0.00	**0.00**	0.00	0.00	0.00	0.00	**0.00**	0.00	0.01	0.01	0.00	**0.01**	0.01	0.01	0.00	0.00	**0.01**	0.01	0.01	0.01	0.01	**0.01**
Isorhamnetin-3-*O*-glucoside	0.21	0.3	0.18	0.16	**0.21**	0.24	0.21	0.27	0.26	**0.25**	0.2	0.33	0.35	0.28	**0.29**	0.14	0.14	0.3	0.17	**0.19**	0.13	0.15	0.12	0.12	**0.13**
Quercetin 3-*O*-glucoside	0.02	0.03	0.02	0.03	**0.03**	0.02	0.02	0.02	0.01	**0.02**	0.02	0.03	0.03	0.02	**0.03**	0.01	0.01	0.03	0.01	**0.02**	0.01	0.01	0.01	0.01	**0.01**
Dihydroquercetin 3-*O*-rhamnoside	0.00	0.00	0.00	0.00	**0.00**	0.00	0.00	0.00	0.00	**0.00**	0.00	0.00	0.00	0.00	**0.00**	0.01	0.01	0.00	0.00	**0.01**	0.01	0.01	0.01	0.01	**0.01**
**Flavonols total**	**0.34**	**0.62**	**0.32**	**0.37**	**0.41**	**0.5**	**0.37**	**0.51**	**0.52**	**0.47**	**0.36**	**0.66**	**0.7**	**0.5**	**0.55**	**0.29**	**0.28**	**0.55**	**0.27**	**0.34**	**0.25**	**0.29**	**0.24**	**0.23**	**0.25**
Procyanidin type B	2.84	2.93	2.98	3.08	**2.96**	2.84	3.00	3.02	2.55	**2.85**	3.37	3.35	3.34	2.05	**3.03**	2.84	2.98	2.68	2.79	**2.82**	2.84	3.00	2.93	1.78	**2.64**
Procyanidin type B	3.06	2.98	2.50	2.74	**2.82**	3.66	3.53	3.48	2.42	**3.27**	3.56	3.29	3.06	2.02	**2.98**	3.06	2.50	3.89	2.92	**3.09**	3.66	3.53	2.98	2.07	**3.06**
(+) catechin	2.65	2.33	1.63	2.50	**2.28**	0.29	0.21	0.20	0.19	**0.22**	0.45	0.61	0.75	0.37	**0.55**	0.34	0.62	0.24	0.41	**0.40**	0.34	0.10	0.60	0.21	**0.31**

Procyanidin type A	4.32	4.08	4.18	4.85	**4.36**	3.45	3.87	3.89	2.54	**3.44**	4.45	3.84	3.8	4.46	**4.14**	4.32	4.18	4.02	3.55	**4.02**	3.45	3.87	4.08	2.32	**3.43**
Procyanidin type A	1.07	0.93	0.57	0.88	**0.86**	0.94	0.85	0.75	0.68	**0.81**	0.74	0.70	0.65	0.42	**0.63**	1.07	0.57	1.06	0.95	**0.91**	0.94	0.85	0.93	0.55	**0.82**
(-) epicatechin	0.95	0.93	0.65	0.57	**0.78**	0.61	0.27	0.21	0.25	**0.34**	0.51	0.14	0.12	0.09	**0.22**	0.11	0.06	0.07	0.05	**0.07**	0.12	0.27	0.08	0.10	**0.14**
Epicatechin gallate	14.13	12.59	11.91	11.76	**12.60**	13.1	12.51	12.55	13.65	**12.95**	13.27	13.13	13.12	8.04	**11.89**	13.55	13.45	12.22	11.97	**12.80**	12.07	13.00	13.88	7.92	**11.72**
**Flavan-3-ols total**	**29.02**	**26.77**	**24.42**	**26.38**	**26.64**	**24.89**	**24.24**	**24.1**	**22.28**	**23.87**	**26.35**	**25.06**	**24.84**	**17.45**	**23.42**	**25.29**	**24.36**	**24.18**	**22.64**	**24.11**	**23.42**	**24.62**	**25.48**	**14.95**	**22.11**
Trans-resveratrol	0.45	0.43	0.41	0.49	**0.45**	0.46	0.38	0.44	0.35	**0.41**	0.34	0.45	0.51	0.56	**0.47**	0.67	0.58	0.37	0.43	**0.51**	0.62	0.56	0.68	0.38	**0.56**
Cis-resveratrol	0.02	0.01	0.01	0.01	**0.01**	0.04	0.01	0.04	0.03	**0.03**	0.01	0.03	0.01	0.01	**0.02**	0.13	0.09	0.05	0.04	**0.08**	0.05	0.02	0.13	0.04	**0.06**
Trans-piceid	1.46	1.16	1.03	0.83	**1.12**	1.25	0.71	1.21	0.92	**1.02**	0.49	1.67	1.51	1.75	**1.36**	1.94	1.41	0.47	0.48	**1.08**	1.78	1.45	1.84	1.53	**1.65**
Cis-piceid	0.26	0.23	0.34	0.39	**0.31**	0.23	0.30	0.21	0.27	**0.25**	0.30	0.24	0.28	0.17	**0.25**	0.44	0.33	0.27	0.21	**0.31**	0.27	0.28	0.3	0.17	**0.26**
**Stilbenes total**	**2.19**	**1.83**	**1.79**	**1.72**	**1.88**	**1.98**	**1.40**	**1.90**	**1.57**	**1.71**	**1.14**	**2.39**	**2.31**	**2.49**	**2.08**	**3.18**	**2.41**	**1.16**	**1.16**	**1.97**	**2.72**	**2.31**	**2.95**	**2.12**	**2.52**
**TOTAL**	**145.80**	**134.00**	**110.90**	**90.90**	**120.40**	**87.50**	**78.50**	**69.90**	**60.00**	**74.00**	**112.70**	**101.40**	**87.40**	**66.30**	**91.90**	**101.10**	**87.30**	**81.40**	**73.00**	**85.70**	**110.70**	**114.20**	**112.80**	**70.80**	**102.10**
